# Differential Effects of Litter Size and Within-Litter Birthweight on Postnatal Traits of Fatty Pigs

**DOI:** 10.3390/ani10050870

**Published:** 2020-05-17

**Authors:** Marta Vázquez-Gómez, Consolacion Garcia-Contreras, José Luis Pesantez-Pacheco, Laura Torres-Rovira, Ana Heras-Molina, Susana Astiz, Cristina Óvilo, Beatriz Isabel, Antonio Gonzalez-Bulnes

**Affiliations:** 1 Departamento de Producción Animal, Facultad de Veterinaria, Universidad Complutense de Madrid, Ciudad Universitaria s/n, 28040 Madrid, Spain; martavazgomez@gmail.com (M.V.-G.); bisabelr@ucm.es (B.I.); 2 Departamento de Mejora Genética Animal, Instituto Nacional de Investigación y Tecnología Agraria y Alimentaria (INIA), Avda Pta. de Hierro s/n, 28040 Madrid, Spain; congarcon@gmail.com (C.G.-C.); ovilo@inia.es (C.Ó.); 3 Departamento de Reproducción Animal, Instituto Nacional de Investigación y Tecnología Agraria y Alimentaria (INIA), Avda Pta. de Hierro s/n, 28040 Madrid, Spain; jose.pesantez@ucuenca.edu.ec (J.L.P.-P.); torrerovi@gmail.com (L.T.-R.); andelash@ucm.es (A.H.-M.); astiz.susana@inia.es (S.A.); 4 Escuela de Medicina Veterinaria y Zootecnia, Facultad de Ciencias Agropecuarias, Universidad de Cuenca, Avda. Doce de Octubre, Cuenca 010220, Ecuador

**Keywords:** developmental programming, intrauterine growth restriction, pig, postnatal growth, prolificacy

## Abstract

**Simple Summary:**

The current work evaluated the relative effects of the number of piglets in the litter of origin (high vs. low litter size) and the birthweight (low (LBW) vs. normal (NBW) in large litters) on postnatal performance and quality of carcasses and meat in purebred Iberian pigs. The results indicated that NBW piglets born in large litters had disparities in developmental patterns in weight and size, back-fat deposition, and fatty acid composition of viscerae compared to NBW piglets from small litters, which again were different from those found in LBW piglets when compared to their NBW counterparts. However, both growth patterns were altered and might indicate previous phases of metabolic disorders.

**Abstract:**

Fatty pigs are characterized by a thrifty genotype, adapted to harsh environments based on changes in metabolism and energy saving. Thus, we hypothesized that feto-maternal energy partitioning in large litters might have postnatal effects that might be independent of intrauterine growth restriction (IUGR) processes. Hence, the current work reported the influence of two effects on postnatal performance and carcass and meat quality of purebred Iberian pigs: (a) the effects of the number of piglets in the litter (high vs. low litter size), and (b) the effects of birthweight (low (LBW) vs. normal (NBW)) in large litters. The results confirmed that NBW piglets born in large litters had differences in developmental patterns of weight, back-fat deposition, and fatty acid (FA) composition when compared to NBW piglets from small litters. These results were different from those found in LBW piglets when compared to their NBW counterparts, which showed an initial asymmetrical growth and altered muscle FA composition at slaughtering. The assessment of FA composition indicated better metabolic status in NBW piglets from large litters than in LBW piglets. These data support the concept that the prenatal environment, even when the individual may cope with it, inescapably affects postnatal life.

## 1. Introduction

There is currently an increasing demand for high-quality dry-cured products from traditional pig breeds like the Iberian breed. Such demands are transforming the traditional extensive management of this animal into more intensive systems, by adopting management strategies from modern breeds and selecting lines with higher prolificacy to improve farm profitability.

Traditionally, the Iberian pig is characterized by lower prolificacy than modern commercial breeds, with around 6–7 piglets per litter [1,2] due to its small uterus and therefore limited uterine capacity [3,4,5]. Currently, the litter size in some farms has risen to around 9–9.5 piglets, which increases the occurrence of intrauterine growth restriction (IUGR) processes and, subsequently, the incidence of neonates with low birthweight (LBW).

The problems derived from LBW have been widely studied in commercial lean pigs [6,7], especially in large litters [8,9,10]. The pork market requires homogeneity in body weight and quality traits of carcasses and meat within batches [11], and such traits are affected in LBW animals when compared to normal birthweight (NBW) individuals [12,13,14,15]. Firstly, the appearance of LBW pigs causes a lack of homogeneity within litters and feedlots. Second, LBW piglets show higher morbidity and mortality, and lower growth potential, lower feed efficiency, and lower meat yield than their NBW littermates [13,16,17]. Moreover, LBW piglets may modify their physiology and metabolism via prenatal programming in response to the inadequate intrauterine environment, whether of maternal or placental origin [15,18]. After birth, these individuals are predisposed to excess adiposity as an adaptive mechanism for energy storage and survival in the inadequate postnatal environment expected, so carcass yields and meat quality are affected [13,15].

In the case of the Iberian pig, there is scarce information about the effects of litter size and birthweight on postnatal development. There have been only a couple of studies using crossbred Iberian × Duroc individuals [19,20], which support a influence similar to that of lean breeds of prenatal conditions and birthweight on postnatal development and meat and carcass quality of offspring under farm conditions. Moreover, birthweight is an even more critical subject of study for productive traits in traditional pigs, because the effects increase with offspring age and traditional pigs are characterized by longer cycles than lean pigs. However, there have been no previous studies on purebred Iberian individuals, which are used for the production of the most expensive dry-cured products. The Iberian pig is characterized by a *thrifty genotype*, adapted to harsh environments, which includes changes in genes and pathways driving metabolism and energy saving [21,22,23]. Thus, we hypothesized that the energy partitioning between sow and fetuses (more dramatic in large litters) might have some effects on postnatal development independent of those due to the IUGR processes. Hence, the current work reports the results of two studies that aimed to determine: (a) the birthweight-independent effects of the number of piglets in the litter of origin (high vs. low litter size) and (b) the effects of birthweight (LBW vs. NBW in large litters) on postnatal performance and carcass and meat quality of purebred Iberian pigs.

## 2. Materials and Methods 

### 2.1. Animals, Management, and Ethics Statements

Two experiments, involving 110 purebred Iberian piglets selected from 24 primiparous Iberian sows (a total of 197 piglets born, without stillbirths) from the Retinto strain of this breed, were developed for this study. These sows were genotyped for purity of breed by determining polymorphisms for *LEPR* gene (homozygosity for *LEPRc.1987T*) by pyrosequencing, as previously described [24]. All the animals, sows and piglets, with no evidence of health problems and adequate pathogen monitoring reports, were housed and managed at INIA animal facilities in agreement with local, national, and European requirements for scientific procedures and animal establishments according to the EU Directive regarding the protection of animals used for scientific purposes (2010/63/UE).

At the beginning of each trial, all of the females weighed around 130 kg and had a mean back-fat depth of around 40 mm. These females were selected, after pregnancy diagnosis on Day 35 of gestation, from a group of animals that were inseminated with purebred Iberian semen after estrus synchronization with altrenogest (Regumate^®^, MSD, Boxmeer, The Netherlands). Sows were allocated to collective outdoor pens with around 7 m^2^ of surface area per animal until one week before expected parturition, after which these females were housed in single indoor pens of 5.49 m^2^ until piglets were weaned. The collective pens had individual feeders so, during pregnancy, each sow had her own diet consisting of 2 kg of standard grain-based food diet with mean values of 13.0% crude protein, 2.8% fat, and 3.00 Mcal/kg metabolisable energy. From farrowing to weaning, average daily feed intake was increased to 3.5 kg per sow. Further information on diets is provided in Supplementary File S1. Water was provided ad libitum during all periods.

After weaning, all the piglets were housed, with males and females separated, in collective outdoor pens and fed with one of two standards diets adapted to age intervals (26–60 and 60–180 days old). From the first month after weaning, the piglets were fed a standard diet with mean values of 18% crude protein, 4.5% fat, and 3.35 Mcal/kg of metabolizable energy; average daily feed intake was 0.5 kg. From 60 to 120 days of age, the piglets were fed a diet containing mean values of 15.1% crude protein, 2.8% fat and 3.08 Mcal/kg of metabolizable energy; the amount of food offered and therefore average daily feed intake were recalculated with age, from 1 to 2.5 kg, to fulfil daily maintenance requirements. Further information on diets is provided in Supplementary File S1. Water was provided *ad libitum* during all periods.

The experimental procedures were assessed and approved by the INIA Committee of Ethics in Animal Research and subsequently by the competent regional authority (report PROEX114/16), according to the Spanish Policy for Animal Protection (RD 53/2013), which meets the European Union Directive 2010/63/UE on the protection of research animals.

### 2.2. Experimental Design

The selection of the piglets for each experiment was done by prioritizing the LITTER-SIZE experiment (first trial) in order to exclude NBW pigs with extreme birthweight and size. Second, LBW and NBW piglets were selected from the same litters for the BIRTH-WEIGHT experiment (second trial). The remaining piglets were only maintained until weaning to avoid differences in the productive condition due to litter size during lactation.

The first experiment (litter size) aimed to study the effects of the number of piglets in the litter of origin (high vs. low litter size), independently of birthweight, and included 54 piglets from small (<8 piglets/litter; 12 females and 12 males) and large litters (≥8 piglets/litter; 15 females and 15 males). In order to avoid effects due to birthweight, the 54 piglets selected were to be similar in weight between both groups and representative of the average weight and size of the breed (i.e., all of them were NBW), and, to avoid effects of milk availability during lactation, underwent within-group fostering in order to equalize the number of piglets among sows.

The second experiment (birthweight) aimed to study the effects of birthweight and included 18 LBW piglets (9 females and 9 males) and 38 NBW piglets (19 females and 19 males) from large litters (≥8 piglets/litter). The threshold for LBW was a birthweight more than one standard deviation below the average birthweight, adjusted by sex [19,25,26]. In all the piglets of both experiments, sex and number of littermates were recorded at birth. The same data and laboratory analyses, described below, were recorded and carried out in both experiments. At weaning, a total of 44 piglets representative of mean body weight and size for their groups were sampled to determine characteristics of the carcass and the meat (four females and four males from small litters plus six females and six males from large litters in the LITTER-SIZE trial, and four LBW females and four LBW males plus eight NBW females and eight NBW males in the BIRTH-WEIGHT trial).

The remaining 66 piglets were used to determine the effects of litter size and birthweight on juvenile development (patterns of growth and fattening and metabolic traits) and quality of carcass and meat at 180 days old (8 females and 8 males from small litters plus 9 females and 9 males from large litters in the LITTER-SIZE trial, and 5 LBW females and 5 LBW males plus 11 NBW females and 11 NBW males in the birthweight trial). However, one male from a large litter and one female from a small litter, and two NBW females died due to accidents not related to the experimental design.

### 2.3. Assessment of Patterns of Growth and Fattening during the Juvenile Period

Body weight and size (trunk length from the nape of the neck to the tail, and abdominal and thoracic circumferences) were measured at birth and again at 15 and 25 days old (when weaning was performed) to characterize the early postnatal performance. Subsequently, values for body weight and size, subcutaneous back-fat depth, and loin diameter were obtained twice monthly for the first three months after weaning (45, 60, 75, and 90 days old) and monthly after that (120, 150 and 180 days old) to characterize the postnatal performance. Body weights were used to calculate intermediate and total average daily weight gain (ADWG) and fractional growth rates (weight gained per day per starting weight; defined by Hansen and co-workers [27]) during the period of study. Subcutaneous back-fat depth (divided into outer and inner layers) and loin diameter were measured using a SonoSite S-Series ultrasound machine with a multifrequency linear array probe (5–8 MHz; SonoSite Inc., Bothell, WA, USA) at the P2 point of the last rib head.

### 2.4. Assessment of Metabolic Traits

Indexes of lipids and glucose metabolism were determined for piglets sampled at weaning (25 days old) and then at 120, 150, and 180 days old. Blood samples were drawn between 9:00 and 10:00 a.m. after a fasting period of around 18 hours, as previously described [28], using heparin vacuum tubes (Vacutainer^®^ Systems Europe, Becton Dickinson, Meylan, France) from either the external jugular vein at 120 and 150 days old, or from the orbital sinus at 180 days old for animal welfare, as fattening of the neck made it difficult to get samples from the jugular. Samples were immediately centrifuged at 1500× *g* for 10 min, and the plasma was separated and stored at −20 °C until it was assayed. Plasma concentrations of parameters indicative of lipids (total cholesterol, high-density lipoprotein cholesterol (HDL-c), low-density lipoprotein cholesterol (LDL-c), and triglycerides) and glucose metabolism (glucose and fructosamine) were measured using a clinical chemistry analyzer (Saturno 300 plus, Crony Instruments SRL, Rome, Italy), according to the manufacturer’s instructions.

### 2.5. Assessment of Body Composition and Tissue Sampling at 25 and 180 Days Old

At 25 and 180 days old, pigs were sequentially euthanized by stunning and exsanguination in compliance with RD53/2013 standard procedures. Immediately, weights of head, carcass, and total and individual viscerae (adrenal glands, brain, heart, intestine, kidneys, liver, lungs, pancreas, and spleen) were determined. The ratios of carcass and viscera weight to body weight were also calculated. Afterwards, samples of brain, liver (the right lateral lobe), muscle (gluteus medius (GM) and longissimus dorsi (LD) at the level of the last rib), and subcutaneous back-fat were obtained and stored at −20 °C to determine fat percentage and fatty acid (FA) composition. A second sample of LD muscle was used on the day of sampling to assess drip-loss capacity. The carcass ratio and data from muscles were used to evaluate carcass and meat quality.

### 2.6. Assessment of Fatty Acid Composition in Diet and Pigs at 25 and 180 Days Old

Fatty acids in the feed were identified, after extraction and methylation, using a gas chromatograph (Hewlett Packard HP-6890, Palo Alto, CA, USA) with a capillary column (HP-Innowax, 30 m × 0.32 mm i.d., and 0.25 µm polyethylene glycol-film thickness and a flame ionization detector [29]). Data are detailed in Supplementary File S1.

The lipids from brain, liver, and muscle (intramuscular fat; IMF) were extracted (expressed as a dry matter percentage [30] and fractionated into the main lipid fractions: neutral lipids (triglycerides) and polar lipids (phospholipids) [31]). Finally, FAs were methylated and identified [32]. Fatty acids of subcutaneous back-fat were also separately analyzed in outer and inner layers. From individual FA values, proportions of saturated, monounsaturated, and polyunsaturated FA (SFA, MUFA, and PUFA), the unsaturated index (UI), and the sum of total n−3 FA (∑n−3) and ∑n−6 FA and its ratio (∑n−6/∑n−3) were calculated [33]. Moreover, the activity of stearoyl-CoA desaturase enzyme 1 (SCD1) was estimated using the desaturation indexes, ratios of C18:1/C18:0 and MUFA/SFA [34].

### 2.7. Statistical Analyses

Data were analyzed using the SAS version 9.4 (SAS Institute Inc., Carry, NC, USA). Dependent variables at 180 and 25 days old (weight, ADWG, body, back-fat and loin measures, FA profile, body composition, and metabolism indexes) were assessed using two-way ANOVA in a general linear model in each independent trial. The LITTER-SIZE trial included sex (female/male) and litter size (<8/≥8 piglets/litter) effects and interactions. The BIRTH-WEIGHT trial included sex and birthweight (LBW/NBW) effects and interactions. Variables with changes over time (all weights, ADWGs, metabolism indexes, and body, back-fat, and loin measures) were also assessed using a repeated-measures ANOVA with the Greenhouse–Geisser correction in each independent trial, using the same fixed factors described for the previous analyses plus time and its interactions. When the interaction was significant, litter size or birthweight were studied within sex. The pig was the experimental unit, and statistical significance was accepted from *p* < 0.05. All the significant results are expressed as mean ±SD in the manuscript and supplementary tables.

## 3. Results

### 3.1. Body Weight and Size at Birth and during the Early Postnatal Period (Lactation)

**Figure 1:**
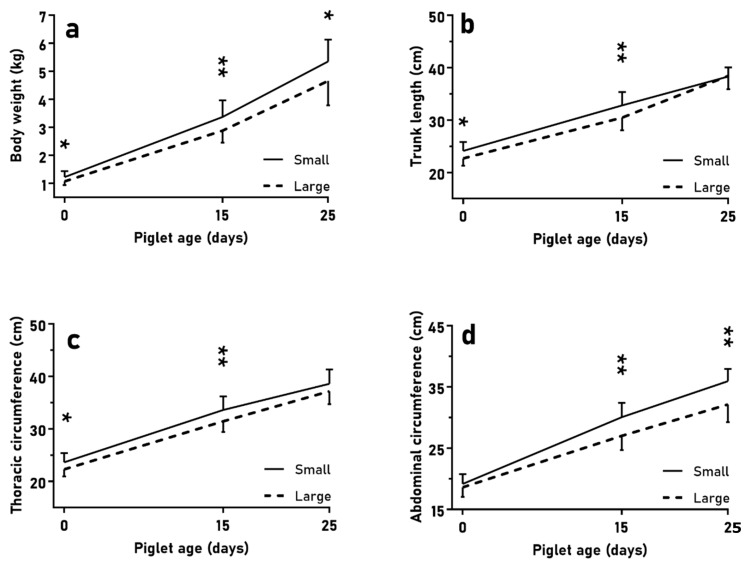
Mean values (±SD) of body weight (**a**), trunk length (**b**), and thoracic and abdominal circumferences (**c**,**d**) over the course of lactation when comparing normal birth-birth (NBW) piglets from small (*n* = 24) and large litters (*n* = 30). Asterisks indicate significant differences between groups (* *p* < 0.05, ** *p* < 0.01).

The first trial (LITTER-SIZE) showed a mean of 4.8 ± 1.5 piglets in small litters and 8.8 ± 1.0 piglets in large litters, without significant differences regarding sex distribution (*p* > 0.10). At birth, piglets selected from small litters showed higher body weight, trunk length, and thoracic circumference than piglets from large litters (Figure 1; *p* < 0.05 for all). The ADWG during lactation was greater in piglets from small litters than in their counterparts from large litters (0–25 days old, 158 ± 22 vs. 139 ± 30 g/d; *p* < 0.05), but there were no differences in fractional growth rate ((*p* > 0.10); further data in Supplementary File S1). Hence, at weaning, piglets from small litters had higher body weight and abdominal circumference than piglets from large litters (*p* < 0.05 for all). However, piglets from small litters showed less total back-fat (3.7 ± 0.7 vs. 4.2 ± 1.4 mm; *p* < 0.05).

**Figure 2:**
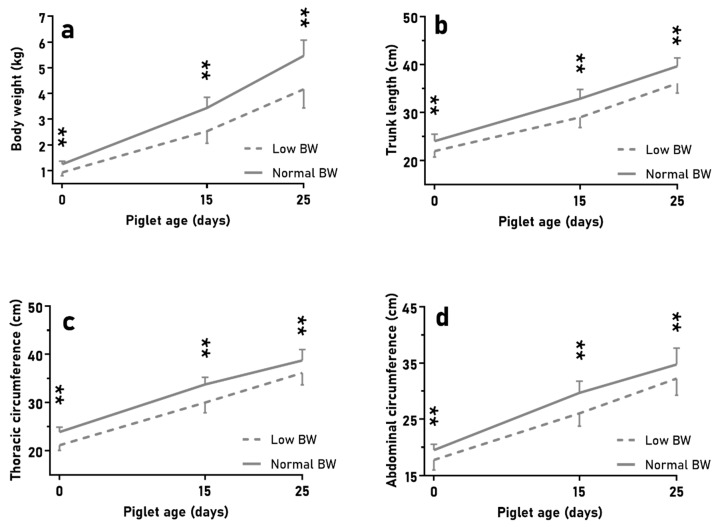
Mean values (±SD) of body weight (**a**), trunk length (**b**), and thoracic and abdominal circumferences (**c**,**d**) over time of lactation when comparing low birth-weight(BW) (*n* = 18) vs. normal BW piglets (*n* = 38) in large litters. Asterisks indicate significant differences (** *p* < 0.01).

The litters used for the second trial (BIRTH-WEIGHT) showed a mean of 9.0 ± 0.8 piglets, with 20.5% of them showing a birthweight lower than one standard deviation below the average birthweight (LBW piglets; around half of them had a birthweight lower than 1 kg). NBW piglets (representative of the breed at 1.3 ± 0.1 kg), as expected due to the study design, showed higher body weight, trunk length, and abdominal and thoracic circumferences than LBW piglets (Figure 2; *p* < 0.01 for all). Subsequently, during lactation, NBW piglets had greater ADWG than LBW piglets (165 ± 19 vs. 128 ± 22 g/d; *p* < 0.05), but there were no differences in fractional growth rate (*p* > 0.10; further data in Supplementary File S1). At weaning, NBW piglets showed higher values for body weight, trunk length, and abdominal and thoracic circumferences (Figure 2) and loin diameter (10.7 ± 1.7 vs. 8.7 ± 1.0 mm; *p* < 0.05) than LBW piglets (*p* < 0.01 for all).

### 3.2. Growth and Fattening during the Juvenile Period

**Figure 3:**
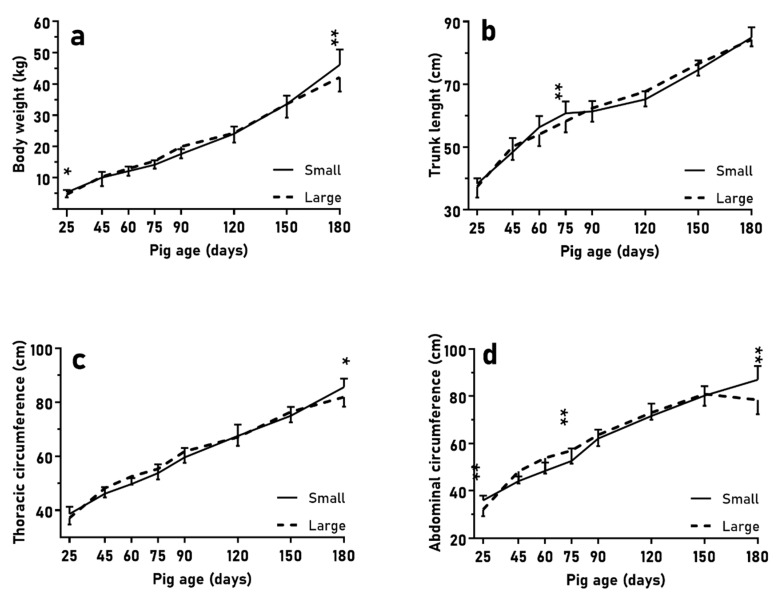
Mean values (±SD) of body weight (**a**), trunk length (**b**), and thoracic and abdominal circumferences (**c**,**d**) over the course of juvenile development (25 to 180 days old) when comparing normal birth-birth (NBW) piglets from small (*n* = 16) and large litters (*n* = 18). Asterisks indicate significant differences (* *p* < 0.05, ** *p* < 0.01).

In the first trial (LITTER-SIZE), the evaluation of body weight (Figure 1a and Figure 3a; *p* < 0.05) and size (Figure 1b,c and Figure 3b–d; *p* < 0.01 for all measurements) from birth to slaughter at 180 days old showed significant differences between pigs from small and large litters, although without a different development in total ADWG or fractional growth rate during the period (*p* > 0.10). Piglets from large litters showed a greater ADWG than their counterparts from small litters during the post-weaning phase (25–75 days old: 221 ± 36 vs. 189 ± 24 g/d; *p* < 0.01) and a greater fractional growth rate from 25 to 75 days old (36.3 ± 6.8 vs. 47.2 ± 6.8 g/kg, *p* < 0.01). The ADWG between 60 and 75 days old was also higher in piglets from large litters (211 ± 75 vs. 139 ± 52 g/d; *p* < 0.01). Consequently, body weight and size (Figure 3) were similar in both groups during this period, and even pigs from large litters showed longer abdominal circumference than pigs from small litters at 75 days old (Figure 3d; *p* < 0.01). Subsequently (from 75 to 180 days old), pigs from small litters had greater ADWG than pigs from large litters (283 ± 56 vs. 245 ± 36 g/d; *p* < 0.05) and greater fractional growth rate than pigs from large litters (20.3 ± 5.1 vs. 16.1 ± 3.5 g/kg, respectively; *p* < 0.05 for both), and reached higher body weight (Figure 3a; *p* < 0.05) and size at 180 days old (Figure 3c,d; *p* < 0.01). There were no differences in body weight and thoracic circumferences from 45 to 150 days old (*p* > 0.10). Interactions with sex were not found either (*p* > 0.10).

**Figure 4:**
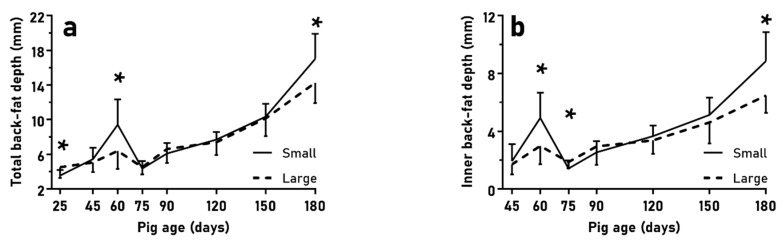
Mean values (±SD) of total and inner-layer back-fat depths (**a**,**b**) over the course of juvenile development (25 to 180 days old) when comparing normal birth-birth (NBW) piglets from small (*n* = 16) and large litters (*n* = 18). Asterisks indicate significant differences (* *p* < 0.05).

The assessment of subcutaneous back-fat depth evolution showed differences for total depth and inner-layer depth betweenn pigs from small litters and pigs from large litters (Figure 4; *p* < 0.01 for both), with significantly higher values in pigs from small litters at 60 and 180 days old (*p* < 0.05, for both), but higher values in pigs from large litters at 75 days old (*p* < 0.05, for both). On the other hand, the assessment of loin diameter also showed higher values in pigs from small litters than in pigs from large litters at 150 days old (20.6 ± 2.3 vs. 18.6 ± 3.3 mm; *p* < 0.05), without sex-related differences (*p* > 0.10). There were no differences in the outer layer depth of back-fat (*p* > 0.10), nor in loin diameter at other time points (*p* > 0.10). 

**Figure 5:**
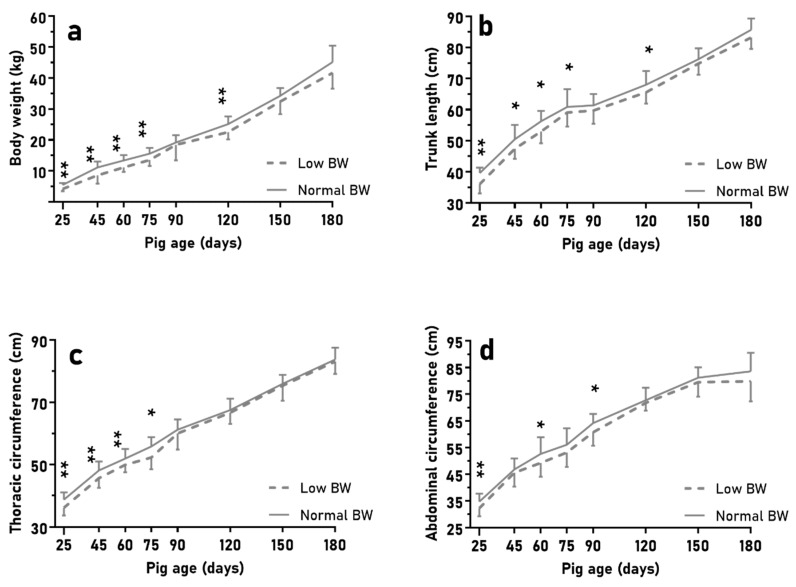
Mean values (±SD) of body weight (**a**), trunk length (**b**), and thoracic and abdominal circumferences (**c**,**d**) over the course of juvenile development (25 to 180 days old) when comparing low birth-weight(BW; (*n* = 10) vs. normal BW piglets (*n* = 22). Asterisks indicate significant differences (* *p* < 0.05, ** *p* < 0.01).

In the second trial (BIRTH-WEIGHT), NBW and LBW pigs had similar ADWG without differences (*p* > 0.10). Pigs with LBW only showed greater fractional growth rates from 25 to 75 days old and overall (46.3 ± 6.5 vs. 39.5 ± 8.9 g/kg, *p* < 0.05; and 242.6 ± 47.8 vs. 191.4 ± 24.9 g/kg, *p* < 0.01; respectively). NBW pigs maintained higher values for body weight and size without different evolution until 150 days old, when no significant differences were found (*p* > 0.10; Figure 5). The loin diameter was also greater in NBW piglets than in LBW piglets, with significant differences at 75 days old (14.2 ± 1.8 vs. 12.7 ± 1.2 mm; *p* < 0.05), but without differences in adiposity (*p* > 0.10) nor at other time points (*p* > 0.1; further data in Supplementary File S1). Again, there were no sex effects (*p* > 0.10).

### 3.3. Metabolic Status at Weaning and during the Juvenile Period

At weaning, piglets from large litters in the LITTER-SIZE trial showed greater fructosamine levels than piglets from small litters (Table 1; *p* < 0.05) and no significant differences in lipid metabolism (*p* > 0.10; Supplementary File S1). On the other hand, in the birthweight trial, LBW piglets showed, when compared to NBW piglets, higher total cholesterol (152 ± 57 vs. 128 ± 23 mg/dl; *p* < 0.05) and LDL-c concentrations (87 ± 56 vs. 67 ± 20 mg/dl; *p* < 0.01), without differences in the glucose metabolism (*p* > 0.10).

After lactation, the assessment of plasma parameters showed no effects of litter size on glucose metabolism (*p* > 0.10). Regarding lipid indexes, pigs from large litters showed greater total cholesterol levels at 120 and 150 days old (Table 1; *p* < 0.05 and *<* 0.01, respectively). Pigs from small litters showed higher plasma LDL-c concentrations than pigs from large litters at 150 days old, but pigs from large litters had higher LDL-c levels than pigs from small litters at 180 days old (*p* < 0.05 for both). On the other hand, there were no differences in metabolic status when comparing NBW and LBW pigs (*p* > 0.10). Finally, no interactions with sex were found in either of the experiments (*p* > 0.10).

### 3.4. Body Composition and Fatty Acid Composition at 25 Days Old

In the first trial (LITTER-SIZE), there were scarcely any differences between piglets from small and large litters; only the relative weight of pancreas to body weight was higher in piglets from large litters (0.11 ± 0.02 vs. 0.13 ± 0.03; *p* < 0.05). There were more profound effects in the birthweight trial because LBW piglets had higher relative weights of head, heart, liver, kidneys, adrenal glands, and pancreas than NBW piglets (Table 2; *p* < 0.01 for all, except *p* < 0.05 for head ratio). No interactions with sex-related effects were found (*p* > 0.10).

The assessment of FA composition showed no effects of LITTER-SIZE of BIRTH-WEIGHT when analyzing the brain (*p* > 0.10), but found significant differences in liver and muscles (further data in Supplementary File S1).

In the case of LITTER-SIZE, the prolificacy affected the polar lipid fraction of the liver but not the neutral fraction. In this way, piglets from small litters showed higher SFA and MUFA levels and lower total PUFA and ∑n−3 and -6 FA concentrations (Table 3; *p* < 0.01 for all, except *p* < 0.05 for SFA and ∑n−3 FA).

In all muscle lipid fractions, the desaturation index C18:1/C18:0 was higher in piglets from small litters than in piglets from large litters (Table 3; *p* < 0.05). There was a significant interaction with sex because females from large litters showed, in the neutral lipid fraction of both muscles (LD and GM), higher SFA concentrations (LD: 38.6 ± 1.0 vs. 34.2 ± 2.0 g/100 g; *p* < 0.05. GM: 41.6 ± 2.3 vs. 35.1 ± 1.0 g/100 g; *p* < 0.01) and lower desaturation indexes (LD: 1.2 ± 0.06 vs. 1.6 ± 0.15 g/100 g; *p* < 0.05; GM: 1.1 ± 0.15 vs. 1.5 ± 0.06 g/100 g; *p* < 0.01) than males from large litters. Finally, the polar lipid fraction of GM also showed greater MUFA levels and desaturation indexes and lower PUFA concentrations in piglets from small litters than in piglets from large litters (*p* < 0.05 for all). 

In the case of BIRTH-WEIGHT, there were differences in all lipid fractions of liver and muscle between LBW and NBW piglets. NBW piglets had higher SFA levels than LBW piglets in both lipid fractions of the liver (Table 4; *p* < 0.01 for neutral and <0.05 for polar lipids). However, LBW piglets showed higher MUFA concentrations and desaturation indexes than NBW piglets in the neutral lipid fraction, and higher Σn−3 and -6 FA concentrations in the polar lipid fraction of liver (*p* < 0.05 for all).

With regards to muscles, NBW piglets had higher ratios of Σ-n6/Σ-n3 FA and C18:1/C18:0 than LBW piglets in the neutral lipid fractions of both muscles (Table 4; *p* < 0.05 for all). However, LBW piglets showed a greater Σ-n6/Σ-n3 FA ratio than NBW piglets in the polar lipid fraction of both muscles (*p* < 0.05 for both). Moreover, in the polar lipid fraction of LD, LBW piglets had higher concentrations of MUFA and lower concentrations of Σn−6 FA than NBW piglets (*p* < 0.05 for both). LBW piglets also showed higher humidity in the LD muscle than NBW piglets (*p* > 0.1). Furthermore, there was also a significant interaction with sex, since NBW females showed a greater Σ-n6/Σ-n3 FA ratio than NBW males in the polar lipid fraction of LD (9.7 ± 0.2 vs. 8.9 ± 0.4; *p* > 0.05).

### 3.5. Body Composition and Fatty Acid Composition at 180 Days Old

In the LITTER-SIZE trial, there were no significant differences in carcass weight between pigs from small and large litters at 180 days old (32.5 ± 4.3 vs. 30.0 ± 3.4 kg ; *p* > 0.1).On the other hand, there were no differences in the carcass weight and ratio between NBW and LBW pigs in the birthweight trial (*p* > 0.10). However, NBW pigs showed higher relative weights of intestine and kidneys to body weight than LBW pigs (intestine: 10.2% ± 1.2% vs. 10.9% ± 1.8%, kidneys: 0.31% ± 0.03% vs. 0.35% ± 0.04%; *p* < 0.05 for both).

There were differences between pigs from small and large litters in the LITTER-SIZE trial when assessing the FA composition of brain and liver. For both viscerae, ratios of Σn−6/Σn−3 FA from neutral and polar lipid fractions were greater in pigs from small litters than in pigs from large litters (Table 5; *p* < 0.01 for liver and *p* < 0.05 for brain). Pigs from small litters also showed higher Σn−6 FA levels than pigs from large litters in the neutral lipid fraction of brain (*p* < 0.05). Moreover, pigs from large litters had greater amounts of Σn−3 FA in both lipid fractions of liver (*p* < 0.01 for polar and *p* < 0.05 for neutral lipids) and a higher desaturation index (C18:1/C18:0; *p* < 0.01) in the polar lipid fraction of brain than pigs from small litters. Finally, there were again sex-related effects, since the ratio Σn−6/Σn−3 FA was greater in the polar lipid fraction of liver in males than in females from small litters (7.5 ± 0.3 vs. 6.7 ± 0.4; *p* < 0.01).

Conversely, there were no differences in main proportions and indexes of FAs in either viscera and back-fat between NBW and LBW pigs in the BIRTH-WEIGHT trial (*p* > 0.10; further data in Supplementary File S1).

In the LITTER-SIZE trial, the assessment of FA composition in the subcutaneous fat showed that both back-fat layers had greater levels of PUFA, both Σn−6 and Σn−3 FA, in pigs from large litters than in pigs from small litters (Table 6; *p* < 0.05 for all). Pigs from large litters also had a higher desaturation index (C18:1/C18:0) and lower SFA levels in the outer layer (*p* < 0.01 for both).The assessment of FA composition in muscle showed that pigs from small litters had greater fat content than pigs from large litters in LD muscle (*p* < 0.05), without differences in GM muscle (*p* > 0.10). Pigs from large litters showed lower SFA levels and higher desaturation indexes than pigs from small litters in the neutral lipid fractions of both muscles (*p* < 0.01 for all, except *p* <0.05 for SFA of GM). Pigs from large litters also had greater MUFA and Σn−3 FA concentrations in the neutral lipid fraction of LD, and a lower Σn−6/Σn−3 ratio in its polar lipid fraction (*p* < 0.01 for all, except in polar fraction where *p* < 0.05).

Moreover, the desaturation index C18:1/C18:0 in the neutral lipid fraction of LD was higher in females than in males in the case of large litters (5.4 ± 0.5 vs. 4.2 ± 0.4; *p* < 0.01). Assessing the neutral lipid fraction of GM, pigs from large litters showed greater PUFA concentrations, both for Σn−6 and Σn−3 FA, and higher Σn−3 FA levels than pigs from small litters (Table 6; *p* < 0.05 for all, except *p* < 0.01 for PUFA). Finally, pigs from small litters had lower SFA concentrations and higher MUFA concentrations and desaturation indexes in the polar lipid fraction of GM than pigs from large litters (*p* < 0.01 for all).

In the BIRTH-WEIGHT trial, the amount of IMF in the case of the GM was higher in LBW pigs than in NBW (20.9 ± 4.0 vs. 17.7 ± 3.3% in dry matter; *p* < 0.05); however, there were no differences when assessing the IMF of LD (*p* > 0.10). Differences in FA composition were mainly found in the neutral lipid fraction of LD muscle; LBW pigs had greater MUFA concentrations and desaturation indexes (Table 7; *p* < 0.05 for all)and lower SFA levels and Σn−6/Σn−3 FA ratio than NBW pigs (*p* < 0.05 for both). No interactions with sex were found in main proportions and indexes of FA (*p* > 0.10).

## 4. Discussion

The results of the present study indicated that the effect of a restricted prenatal environment on offspring phenotype and performance goes beyond occurrence of intrauterine growth restriction (IUGR) and subsequent lowBW (LBW) with its inherent complications. Piglets with normal BW (NBW) but born within large litters (larger than eight piglets), which implies a lower availability of uterine space and therefore of nutrients and oxygen, had differences in developmental patterns, fat deposition, and FA composition when compared to NBW piglets from small litters. These data support the notion that the prenatal environment, even when the individual may cope with it, inescapably affects postnatal life.

The current study included two different trials aiming to determine, in animals of the same breed maintained under same environment and management conditions, the relative weight of the effects of either BIRTH-WEIGHT (by comparing NBW and LBW piglets) or the LITTER-SIZE (availability of uterine space and resources) independently of the birthweight (by comparing NBW piglets from large and small litters).

The results of the experiment aiming to determine the effects of BIRTH-WEIGHT showed, as expected, similar results to previous studies on both commercial lean strains [9,13] and traditional fatty pigs [19,20,35]. In brief, in agreement with the experimental design, the piglets selected as representative of LBW showed, at birth, significantly lower body weight and size (trunk length and abdominal and thoracic circumferences) than the piglets selected as NBW. These differences were maintained or even increased during the early postnatal period, because NBW piglets showed higher ADWG and better muscle development, in agreement with results previously reported for lean breeds [36]. Hence, LBW piglets remained smaller and lighter at weaning, showing higher relative weights of head, heart, liver, kidneys, adrenal glands, and pancreas than NBW piglets (i.e., evidenced asymmetrical growth patterns), which are all signs of IUGR [37].

After weaning, during the juvenile period, NBW pigs remained significantly heavier and larger until 75 days old (i.e., during the post-weaning phase and before the late-growing phase). Afterwards, differences in body weight and corpulence between NBW and LBW pigs were not statistically significant and the relative sizes of carcass and viscerae were ultimately similar in both groups, except for intestine and kidneys, with lower relative weights in LBW than in NBW at 180 days old, these being two of the systems more affected in case of IUGR [38,39]. We also have to note that NBW pigs maintained better muscle development while LBW pigs showed increased intramuscular fat content, despite no differences in subcutaneous or visceral adiposity. These results are in agreement with previous studies in both lean [40,41] and fatty pigs [42,43] and are thought to be consequent to alterations in adipogenesis during prenatal stages [44].

The results of the experiment aiming to determine the influence of LITTER-SIZE showed that piglets from large litters, similarly to LBW piglets in the BIRTH-WEIGHT trial, showed the worst ADWG during lactation and, therefore, the lowest body weight and size at weaning. However, in contrast to the LBW piglets of the BIRTH-WEIGHT trial, piglets from large litters showed higher adiposity than piglets from small litters at weaning,without evidence of asymmetrical growth patterns between groups except in the pancreas. It was not possible to elucidate the metabolic implications of differences in the size of the pancreas under the design of the current study; however, it is known that this organ is one of the most affected in case of prenatal programming, with changes having a prominent role in metabolic disturbances [45,46].

Subsequently, during the postnatal period, piglets from large and small reached similar values of body weight and size in spite of very different patterns of ADWG. Pigs from large litters had a more precocious weight gain, with higher fractional growth rate, during the post-weaning phase (25–75 days old), and even higher adiposity and larger abdominal circumference at 75 days old (abdominal circumference is predictive of carcass and visceral fat, as validated by quantitative dissection [47]). Afterwards, pigs from small litters showed a higher weight gain, with higher ADWG and fractional growth rate, during the late-growing phase (75–180 days old) and reached higher body weight, body size, and adiposity (including higher intramuscular fat content) than piglets from small litters at 180 days old.

The comparison of growth patterns in LBW piglets in the BIRTH-WEIGHT trial and NBW piglets from large litters in the LITTER-SIZE experiment suggested two very different developmental patterns. In brief, LBW piglets were still affected by growth restriction during the post-weaning period (25–75 days old), in spite of developing a higher fractional growth rate than NBW counterparts. They also showed compensatory catch-up growth only during the late-growing period (75–180 days old), as already observed in previous studies in LBW Iberian crossbred pigs [20]. On the other hand, piglets from large litters showed a very early catch-up growth during the post-weaning period (25–75 days old) which slowed down during the late-growing period, when pigs from small litters were initiating their fattening period. There were no major effects of BIRTH-WEIGHT or LITTER-SIZE on metabolic features during postnatal development, except at weaning. At that moment, there were higher concentrations of total and LDL cholesterol in LBW piglets and higher fructosamine concentrations in piglets from large litters. Fructosamine is indicative of precedent glucose availability, so such data may indicate some degree of insulin resistance for attaining compensatory growth [48].

The assessment of FA composition at weaning showed, besides similarities in the developmental pattern during lactation, analogous changes in LBW piglets from the BIRTH-WEIGHT trial and piglets from large litters in the LITTER-SIZE experiment when compared to their respective NBW and small-litter counterparts. In brief, there were no differences in the FA profile of the brain among piglets in both trials. On the other hand, the assessment of the polar lipid fraction of the liver showed a decrease in SFA levels with increases in Σn−3 and Σn−6 FA in both LBW and large-litter piglets; piglets from large litters also showed a decrease in MUFA values. The assessment of the neutral fraction of the liver showed no changes in piglets from large litters, but LBW piglets showed increases in MUFA content and desaturation index, which it is a feature commonly related to metabolic disorders such as obesity and insulin resistance in both humans [34,49] and pigs [50,51]. Similarities at the muscle level were less evident, with only a decrease in the desaturation index of both lipid fractions in LBW and large-litter piglets. Piglets from large litters also showed a decrease in MUFA values and increases in PUFA concentrations in the polar lipid fraction. LBW piglets showed an increase of the Σn−6/Σn−3 FA ratio in the polar fraction, which may confirm previous evidence of metabolic disorders and insulin resistance [52]. Conversely, the assessment of FA composition at 180 days old showed completely different profiles between LBW and large-litter piglets when compared to their NBW and small-litter counterparts. Thus, these groups had different evolutions in their FA composition throughout development, from weaning to 180 days old.

There were no main differences between LBW and NBW pigs in the BIRTH-WEIGHT trial in the FA profile of brain, liver, and subcutaneous fat—results which are very different from previous studies in Iberian pigs [19,20]. However, we should bear in mind that, in contrast to these previous studies, the pigs currently studied were sampled at an earlier age, prior to the fattening period. In the muscle, the FA composition of the polar fraction was also similar in both groups (a foreseeable result, since membrane lipids of the polar fraction are more stable than storage lipids of the neutral fraction [53]). On the other hand, there were significant differences in the neutral fraction, where MUFA content and desaturation index increased in LBW pigs (confirming results found at weaning and therefore predisposition to metabolic disorders in these pigs [34,49]). Conversely, SFA values and the ratio of Σn−6/Σn FA decreased, which is protective against metabolic disorders by diminishing pro-inflammatory status and favoring the action of insulin [54].

On the other hand, there was a plethora of differences in the FA composition of pigs from large and small litters in the LITTER-SIZE experiment. Viscerae (brain and liver) of pigs from large litters showed a decrease in Σn−6/Σn−3 FA ratio in both neutral and polar lipid fractions, which was related to a decrease in Σn−6 FA concentrations in the brain and an increase in Σn−3 levels in the liver. These changes were all protective for the individual. Higher availability of Σn−3 has been found to improve pro-/anti-inflammatory status, insulin function, and physical and mental development during the first years of life [55,56,57]; higher availability of Σn−3 FA is related to higher availability of anti-inflammatory lipid mediators, which reduce pathological risks [58,59]. Higher availability of Σn−6 FA would be related to higher availability of pro-inflammatory lipid mediators [60]. Therefore, an increase of Σn−6/Σn−3 FA ratio in the liver would indicate a pro-inflammatory state related to increased peripheral lipolysis and increased flux of FAs [54].

Assessment of the FA content of triglycerides in the subcutaneous fat showed increases of PUFA, Σn−6, and Σn−3 FA values in both outer and inner layers of pigs from large litters. The outer layer also showed a decrease in the SFA content and an increase in the desaturation index. The same changes (increases of concentrations of PUFA, Σn−6 FA, and Σn−3 FA and desaturation index, and decrease in SFA values) were found in the neutral fraction (triglycerides) of the GM muscle. This finding is logical from the physiological point of view, since FAs are mostly distributed in the neutral fraction (around 70% of total FAs [61]) and, besides that, the polar fraction is more stable than the neutral fraction, as previously discussed [53]. On the other hand, only the increase in the desaturation index and the decrease in SFA values were found in the LD muscle (which were the same changes found in LBW). These differences in the FA profiles between GM and LD muscle were likely related to the content of oxidative muscle fibers [62], because fibers of the GM have a predominantly oxidative metabolism while fibers of the LD have a mainly glycolytic metabolism.The LD muscle also showed a decrease in the ratio of Σn−6/Σn−3 FA, but in the polar fraction (coincidentally with changes in the polar fraction of brain and liver). In the skeletal muscle, the Σn−3 FA content of cellular membranes plays a main role favoring the action of insulin; a high Σn−6/Σn−3 FA ratio would be deleterious to insulin sensitivity [63] and ultimately would result in insulin resistance [52].

Hence, the FA profile found in NBW piglets from large litters was mostly protective for their metabolic health (higher levels of PUFA and Σn−6 FA levels with lower ratios Σn−6/Σn−3 FA and lower content of SFA); only the increase in desaturation indexes was a warning. On the other hand, from a productive point of view, increases in the PUFA content and decreases in the SFA content and Σn−6/Σn−3 FA ratio found in the muscle and subcutaneous fat this imply health and sensorial benefits and therefore enhance consumer acceptance [64,65,66].

## 5. Conclusions

In conclusion, piglets with NBW from large litters, in opposition to LBW piglets, are prone to higher adiposity than piglets from small litters as early as during the suckling period. After weaning, these piglets show a very early catch-up growth after exposure to high-energy feed during the post-weaning period (25–75 days old), which may be related to insulin resistance. Afterwards, during the juvenile period (fed with maintenance diets), the catch-up growth slows down and, based on the FA composition assessment, we inferred a better metabolic status in NBW piglets from large litters than in LBW piglets, independently of the litter size. These results, overall, show clear effects of the litter size on the postnatal development in NBW pigs, which are independent of effects due to LBW.

## Figures and Tables

**Table 1:** Highlighted significant differences (*p* < 0.05) in metabolic parameters between piglets from large and small litters.

Age (Days)	Variables	n	Litter Size	
			Small (<8)	Large (≥8)
25	Fructosamine (mmol/L)	20	284.8 ± 33.3	349.0 ± 38.4
120	Cholesterol (mg/dL)	32	115.8 ± 13.8	103.8 ± 14.2
150	Cholesterol (mg/dL)	32	111.9 ± 16.0	104.7 ± 18.0
	LDL-c (mg/dL)	32	56.1 ± 13.2	49.4 ± 9.9
180	LDL-c (mg/dL)	32	53.50 ± 12.9	55.80 ± 11.5

LDL-c = low-density lipoprotein cholesterol.

**Table 2:** Highlighted significant differences (*p* < 0.05) in relative weight to body weight (percentage) of different organs at 25 days old between piglets with low or normal birthweight.

Component	Birthweight	
	Low	Normal
Head	15.7 ± 2.7	14.3 ± 1.2
Adrenal glands	0.021 ± 0.004	0.017 ± 0.002
Kidneys	0.72 ± 0.12	0.59 ± 0.05
Heart	0.74 ± 0.07	0.63 ± 0.05
Liver	3.04 ± 0.35	2.71 ± 0.16
Pancreas	0.16 ± 0.03	0.11 ± 0.02

*n* = 24.

**Table 3:** Highlighted significant differences (*p* < 0.05) in fatty acid (FA) composition of different tissues between piglets from small or large litters at 25 days old.

Tissue	Lipid Fraction	Variable	Litter Size	
			Small (<8)	Large (≥8)
Liver	Polar	SFA (g/100 g FA)	49.1 ± 2.7	46.5 ± 3.4
		MUFA (g/100 g FA)	16.3 ± 2.2	13.5 ± 1.8
		PUFA (g/100 g FA)	34.6 ± 4.1	40.0 ± 3.7
		Σn−3 (g/100 g FA)	5.3 ± 1.2	6.8 ± 1.4
		Σn−6 (g/100 g FA)	28.9 ± 3.1	32.8 ± 2.4
LD	Neutral	C18:1/C18:0	7.5 ± 0.8	6.8 ± 1.2
	Polar	C18:1/C18:0	1.7 ± 0.1	1.5 ± 0.2
GM	Neutral	C18:1/C18:0	7.7 ± 0.9	6.8 ± 1.2
	Polar	MUFA (g/100 g FA)	20.6 ± 1.7	18.8 ± 1.1
		PUFA (g/100 g FA)	41.3 ± 1.7	43.2 ± 0.6
		MUFA/SFA	0.54 ± 0.05	0.50 ± 0.04
		C18:1/C18:0	1.4 ± 0.1	1.2 ± 0.42

LD = Longissimus dorsi muscle, GM = Gluteus medius muscle. *n* = 20. IMF = intramuscular fat, FA = fatty acids, IF = Intratissue fat, SFA = sum of saturated FA; MUFA = sum of monounsaturated FA, PUFA = sum of polyunsaturated FA.

**Table 4:** Highlighted significant differences (*p* < 0.05) in the fatty acid (FA) composition of different tissues between piglets with normal or low birthweight at 25 days old.

Tissue	Lipid Fraction	Variable	Birthweight	
			Low	Normal
Liver	Neutral	SFA (g/100 g FA)	44.8 ± 3.8	49.0 ± 3.3
		MUFA (g/100 g FA)	22.2 ± 6.8	16.7 2.7
		MUFA/SFA	0.50 ± 0.17	0.34 ± 0.06
		C18:1/C18:0	0.9 ± 0.4	0.5 ± 0.1
	Polar	SFA (g/100 g FA)	45.3 ± 3.0	48.6 ± 3.0
		PUFA (g/100 g FA)	41.9 ± 1.0	35.8 ± 4.4
		Σn−3 (g/100 g FA)	7.4 ± 0.9	5.6 ± 1.3
		Σn−6 (g/100 g FA)	34.0 ± 0.6	29.7 ± 3.2
		Humidity (%)	76.7 ± 1.3	75.3 ± 1.2
LD	Neutral	Σn−6/Σn−3	11.5 ± 1.3	12.1 ± 0.6
		C18:1/C18:0	5.9 ± 0.4	7.5 ± 0.9
	Polar	MUFA (g/100 g FA)	20.7 ± 1.3	23.6 ± 2.1
		Σn−6 (g/100 g FA)	38.3 ± 1.0	35.8 ± 1.8
		Σn−6/Σn−3	10.1 ± 0.6	9.2 ± 0.6
		C18:1/C18:0	1.4 ± 0.1	1.6 ± 0.1
GM	Neutral	Σn−6/Σn−3	10.8 ± 1.4	11.5 ± 0.5
	Polar	Σn−6/Σn−3	10.6 ± 1.1	9.5 ± 0.7
		C18:1/C18:0	1.1 ± 0.2	1.4 ± 0.1

LD = Longissimus dorsi muscle; GM = Gluteus medius muscle. *n* = 24. IMF = intramuscular fat; SFA = sum of saturated FA; MUFA = sum of monounsaturated FA; PUFA = sum of polyunsaturated FA.

**Table 5:** Highlighted significant differences (*p* < 0.05) in fatty acid composition of brain and liver between piglets from small and large litters at 180 days old.

Tissue	Lipid Fraction	Variable	Litter Size	
			Small (<8)	Large (≥8)
Brain	Neutral	Σn−6 (g/100 g FA)	17.0 ± 0.6	16.0 ± 1.0
		Σn−6/Σn−3	1.9 ± 0.1	1.8 ± 0.1
	Polar	Σn−6/Σn−3	1.9 ± 0.1	1.8 ± 0.1
		C18:1/C18:0	1.5 ± 0.1	1.6 ± 0.1
Liver	Neutral	Σn−3 (g/100 g FA)	2.6 ± 0.5	3.3 ± 0.6
		Σn−6/Σn−3	8.3 ± 0.9	7.1 ± 0.8
	Polar	Σn−3 (g/100 g FA)	3.5 ± 0.5	4.4 ± 0.8
		Σn−6/Σn−3	7.0 ± 0.5	6.0 ± 1.1

IMF = intramuscular fat; FA = fatty acids; *n* = 32; SFA = sum of saturated FA; MUFA = sum of monounsaturated FA; PUFA = sum of polyunsaturated FA.

**Table 6:** Highlighted significant differences (*p* < 0.05) in fatty acid composition of back-fat and muscle between piglets from small and large litters at 180 days old.

Tissue	Lipid Fraction/Layer	Variable	Litter Size	
			Small (<8)	Large (≥8)
Back-fat	Outer	SFA (g/100 g FA)	38.8 ± 2.1	35.4 ± 2.7
		PUFA (g/100 g FA)	13.8 ± 1.2	16.4 ± 1.7
		Σn−3 (g/100 g FA)	1.0 ± 0.1	1.2 ± 0.2
		Σn−6 (g/100 g FA)	10.9 ± 1.0	13.1 ± 1.5
		MUFA/SFA	1.3 ± 0.1	1.4 ± 0.2
	Inner	PUFA (g/100 g FA)	15.5 ± 1.1	17.5 ± 1.5
		Σn−3 (g/100 g FA)	1.1 ± 0.1	1.3 ± 0.1
		Σn−6 (g/100 g FA)	12.1 ± 1.2	13.9 ± 1.3
		IMF (%)	17.6 ± 4.9	14.0 ± 3.7
LD	Neutral	SFA (g/100 g FA)	39.0 ± 2.5	36.8 ± 2.1
		MUFA (g/100 g FA)	55.0 ± 2.3	56.3 ± 2.2
		Σn−3 (g/100 g FA)	0.56 ± 0.09	0.64 ± 0.09
		MUFA/SFA	1.4 ± 0.2	1.5 ± 0.1
		C18:1/C18:0	4.0 ± 0.6	4.6 ± 0.7
	Polar	Σn−6/Σn−3	11.7 ± 0.8	11.1 ± 0.7
GM	Neutral	SFA (g/100 g FA)	38.4 ± 2.0	37.2 ± 2.4
		PUFA (g/100 g FA)	8.0 ± 1.3	10.0 ± 1.9
		Σn−3 (g/100 g FA)	0.74 ± 0.10	0.88 ± 0.13
		Σn−6 (g/100 g FA)	7.0 ± 1.1	8.7 ± 1.7
		C18:1/C18:0	4.0 ± 0.5	4.2 ± 0.6
	Polar	SFA (g/100 g FA)	35.6 ± 0.7	36.7 ± 0.7
		MUFA (g/100 g FA)	16.9 ± 1.4	14.7 ± 1.6
		Σn−3 (g/100 g FA)	3.65 ± 0.2	3.9 ± 0.3
		MUFA/SFA	0.48 ± 0.04	0.40 ± 0.05
		C18:1/C18:0	1.3 ± 0.1	1.0 ± 0.2

LD = Longissimus dorsi muscle; GM = Gluteus medius muscle; *n* = 32; FA = fatty acids; IMF = intramuscular fat; SFA = sum of saturated FA; MUFA = sum of monounsaturated FA; PUFA = sum of polyunsaturated FA.

**Table 7:** Highlighted significant differences (*p* < 0.05) in muscle fatty acid composition between pigs with normal and low birthweight at 180 days old.

Tissue	Lipid Fraction	Variable	Birthweight	
			Low	Normal
LD	Neutral	SFA (g/100 g FA)	36.7 ± 2.2	38.5 ± 2.6
		MUFA (g/100 g FA)	57.4 ± 2.1	54.7 ± 1.8
		Σn−6/Σn−3	8.7 ± 0.8	9.6 ± 1.1
		MUFA/SFA	1.6 ± 0.1	1.4 ± 0.1
		C18:1/C18:0	4.7 ± 0.7	4.1 ± 0.6

LD = Longissimus dorsi muscle; FA = fatty acids; *n* = 30; SFA = sum of saturated FA; MUFA = sum of monounsaturated FA.

## Supplementary Materials

The following are available online at https://www.mdpi.com/2076-2615/10/5/870/s1, Supplementary file S1.
